# Electrochemical and Electronic Charge Transport Properties of Ni-Doped LiMn_2_O_4_ Spinel Obtained from Polyol-Mediated Synthesis

**DOI:** 10.3390/ma11050806

**Published:** 2018-05-16

**Authors:** Shuo Yang, Dirk Oliver Schmidt, Abhishek Khetan, Felix Schrader, Simon Jakobi, Melanie Homberger, Michael Noyong, Anja Paulus, Hans Kungl, Rüdiger-Albert Eichel, Heinz Pitsch, Ulrich Simon

**Affiliations:** 1 Institute of Inorganic Chemistry, RWTH Aachen University, 52074 Aachen, Germany; oliver.schmidt@rwth-aachen.de (D.O.S.); felix.schrader@rwth-aachen.de (F.S.); simon.jakobi@ac.rwth-aachen.de (S.J.); melanie.homberger@ac.rwth-aachen.de (M.H.); michael.noyong@ac.rwth-aachen.de (M.N.); 2 Jülich Aachen Research Alliance-JARA, 52428 Jülich, Germany; anj.paulus@fz-juelich.de (A.P.); h.kungl@fz-juelich.de (H.K.); r.eichel@fz-juelich.de (R.-A.E.); 3 Institute for Combustion Technology, RWTH Aachen University, 52056 Aachen, Germany; askhetan@gmail.com (A.K.); h.pitsch@itv.rwth-aachen.de (H.P.); 4 Institute of Energy and Climate Research IEK-9: Fundamental Electrochemistry, Forschungszentrum Jülich, 52425 Jülich, Germany; 5 Institute of Physical Chemistry, RWTH Aachen University, 52074 Aachen, Germany

**Keywords:** Lithium-ion battery, LiNi_0.5_Mn_1.5_O_4_ spinel, DFT calculations, rate capability, electrical conductivity

## Abstract

LiNi_0.5_Mn_1.5_O_4_ (LNMO) spinel has been extensively investigated as one of the most promising high-voltage cathode candidates for lithium-ion batteries. The electrochemical performance of LNMO, especially its rate performance, seems to be governed by its crystallographic structure, which is strongly influenced by the preparation methods. Conventionally, LNMO materials are prepared via solid-state reactions, which typically lead to microscaled particles with only limited control over the particle size and morphology. In this work, we prepared Ni-doped LiMn_2_O_4_ (LMO) spinel via the polyol method. The cycling stability and rate capability of the synthesized material are found to be comparable to the ones reported in literature. Furthermore, its electronic charge transport properties were investigated by local electrical transport measurements on individual particles by means of a nanorobotics setup in a scanning electron microscope, as well as by performing DFT calculations. We found that the scarcity of Mn^3+^ in the LNMO leads to a significant decrease in electronic conductivity as compared to undoped LMO, which had no obvious effect on the rate capability of the two materials. Our results suggest that the rate capability of LNMO and LMO materials is not limited by the electronic conductivity of the fully lithiated materials.

## 1. Introduction

The need to involve renewable energy sources to fulfill the global energy demand necessitates the development of large-scale energy storage systems [1,2]. In the past decades, Li-ion batteries have attracted much attention owing to their high energy density and long cycle life [3,4].

As one of the most promising cathode material candidates, LiMn_2_O_4_ (LMO) spinel offers advantages such as high rate capability, good safety, low cost and nontoxicity [5,6,7,8,9]. However, a significant challenge posed to the usage of this material is the capacity-fading during cycling, especially at elevated temperature [5,6,7,8,9]. To overcome this challenge, several bi- or trivalent cations, such as Co [10], Ni [11], and Fe [12], have been investigated in their role as dopants to partially substitute the Mn in LMO. Among these materials, the Ni-doped LMO spinels, especially the LiNi_0.5_Mn_1.5_O_4_ (LNMO), have been extensively studied [9,13,14].

LNMO offers a theoretical specific discharge capacity of 146.7 mAh/g and a high operating voltage of 4.7 V vs. Li/Li^+^, which leads to a high energy density of approximately 700 Wh/kg [15]. Unlike undoped LMO, where the average oxidation state of Mn is +3.5, the Mn in the LNMO exhibits an oxidation state of +4, which suppresses the Mn dissolution and Jahn–Teller (JT) distortion, and thus leads to enhanced cycling performance [16].

Regarding the ordering of Mn and Ni cations, two possible crystallographic structures have been reported for LNMO: the so-called “disordered” spinel phase with the space group of Fd$\bar{3}$m and the “ordered” spinel phase with the space group of P4_3_32 [17,18,19]. Previous works have revealed that significant amount of short-range Ni/Mn ordering may occur in LNMO materials and the obtained LNMO materials are typically composed of both disordered and ordered phases [20,21,22]. The disordered spinel has been reported to show superior rate performance to the ordered phase [17,18,23]. One of the widely accepted explanations for this is the enhanced electronic conductivity due to the presence of Mn^3+^ in the disordered spinel [24,25].

However, most of the electrical investigations conducted so far are bulk measurements, such as four-probe AC measurements or impedance spectroscopy (IS) measurements, on pressed pellets [26,27]. To densify the pellets, sintering at high temperature is generally required, which can influence the crystallographic structure of LNMO and/or result in formation of secondary phases [28,29]. Moreover, during the bulk measurements, it is challenging to discriminate between bulk conductivity and grain boundary conductivity. Therefore, it is desirable to directly investigate the electrical properties of individual micro- and nanometer-scaled particles with well-defined morphology, as already pointed out by Moorhead-Rosenberg et al. [20], to interrelate these measurements to first principles calculations, and to correlate the electronic charge transport properties with the electrochemical performance.

Such an approach would require the combination of experimental, theoretical, and synthetic procedures, which, to the best of our knowledge, has not been reported yet in this particular field.

Measurements of the electrical conductivity of individual nanometer-scaled particles in a highly flexible manner can be obtained by using a nanorobotics setup in a scanning electron microscope (SEM), which has been successfully applied to different classes of materials and morphologies [30,31,32,33]. By using this setup, we recently performed local electrical transport measurements on single Fe-, Ti-doped and Ru-, Ti-doped LNMO spinel crystals and demonstrated a change in the electrical conductivity depending on the dopant, which was consistent with IS measurements on pressed pellets of the corresponding materials [34].

Small-polaron hopping in LMO has been computationally studied in significant detail [35,36,37]. Although several theoretical studies have reported on the crystal and electronic structure of LNMO materials [38,39], an investigation of the effect of Ni doping on small-polaron conductivity has not yet been performed.

Several synthesis methods, such as solid-state reactions [22], the Pechini method [24], sol-gel method [40], co-precipitation method [41], and polymer-assisted synthesis [41,42,43,44], have been proposed to prepare LNMO with various particle morphologies. Recently, we reported the synthesis of nanostructured single phase LMO spinel via polyol method for utilization as cathode material [45]. During the synthesis, the polyol serves as both solvent and mild reducing agent. As soon as the precipitation starts, the polyol adsorbs instantaneously onto the surface of the as-formed particle nuclei, which limits the particle growth and prevents aggregation, offering remarkable control over the particle size and morphology [8,46,47].

In this paper, we present the polyol-mediated synthesis of LNMO and its characterization by means of powder X-Ray diffraction (pXRD), scanning electron microscopy (SEM), selected area electron diffraction (SAED), energy dispersive X-ray spectroscopy (EDX) as well as electrochemical measurements. The focus of this work is the investigation of the electronic charge transport properties of individual particles of the as-prepared LNMO measured in local electrical transport measurements in comparison to its rate capability. To further study the electrical conductivity of LNMO and to examine the effect of Ni doping of LMO materials on their electrical conductivity, first principles calculations were performed on undoped LMO and LiNi_0.375_Mn_1.625_O_4_ to estimate the barriers for small-polaron hopping. According to our investigations, the as-prepared LNMO exhibited cycling stability and rate capability comparable to those of the LNMO materials reported in the literature. The electrical conductivity of the as-prepared LNMO was about one order of magnitude lower than the undoped LMO. Nevertheless, the difference in electronic conductivity had no effect on the rate capability of the two materials, which suggests that the rate capability of LNMO and LMO materials is not limited by the electronic conductivity of the materials in their fully lithiated state.

## 2. Materials and Methods

### 2.1. Polyol-Mediated Synthesis of LNMO

LNMO particles were synthesized via polyol method adopting a similar procedure as previously described elsewhere for the undoped LMO [45]. LiOH (Merck, Darmstadt, Germany, >98%), Ni(OH)_2_ (Sigma-Aldrich, Steinheim, Germany, 99.2%) and electrolytic manganese oxide (EMD, grade HMR-AF, Tosoh, Tokyo, Japan, 92.5%) were selected as metal precursors. The Li:Mn and Mn:Ni precursor molar ratios were set as 2.12 and 2.77, respectively. As solvent and reducing agent, ethylene glycol (EG, Steinheim, Germany, >99.5%) was used. After the reaction, the acquired precipitates were recovered by centrifuging in 50 mL polystyrene tubes (Carl Roth, Karlsruhe, Germany) using a UniCen MR centrifuge (Herolab, Wiesloch, Germany) with 14,000 rpm for 30 min at 20 °C. They were washed twice with acetone and centrifuged with 5000 rpm for 5 min. Afterwards, the as-synthesized LNMO powders were dried under vacuum at room temperature for 3 h, ball milled and calcined at 250 °C and 800 °C in air. For purposes of comparison, LMO particles were also synthesized according to Yang et al. [45], ball milled and calcined at 250 °C to remove the organic residual and then at 800 °C for the formation of the spinel phase. A more detailed description of the sample preparation can be found elsewhere [45].

### 2.2. Physicochemical Characterization

Powder XRD measurement was conducted at room temperature using a STADI P diffractometer (Stoe & Cie GmbH, Darmstadt, Germany) operating in transmission mode with Cu K_α1_ radiation (λ = 1.54059 Å). The acquired powder XRD data were processed with the software WinXPOW Version 1.06 (Stoe & Cie GmbH).

The particle morphology of the as-prepared LNMO was analyzed using a high resolution field emission scanning electron microscope (FE-SEM, Leo Supra 35 VP (Carl Zeiss AG, Oberkochen, Germany). The element distribution of Mn and Ni in the LNMO particles was characterized by EDX. To verify the chemical composition of individual particles, EDX measurements were performed in a Libra 200 field emission transmission electron microscope (FE-TEM, Carl Zeiss AG, Oberkochen, Germany) operated at 200 keV equipped with an XFlash 5030 EDX detector (Bruker, Billerica, MA, USA). Prior to the measurements, the sample was deposited on TEM grids with SiO_2_ windows (SiO_2_ thickness: 20 nm, SiMPore Inc., Rochester, NY, USA).

The local electrical transport measurements were performed in the FE-SEM mentioned above. Prior to the measurements, the as-prepared LNMO particles were deposited on silicon wafers with a SiO_2_ layer of 100 nm thickness. During the measurements, 19 LNMO particles were addressed individually with two homemade metalized atomic force microscopy (AFM) tips (ATECNC, Nanosensors) via a nanorobotics system (Klocke Nanotechnik, Aachen, Germany). These tips were uniformly coated with Pt/Ir alloy (80% Pt, 20% Ir) by RF sputtering (0.017 mbar Ar/40 W) and exhibited a tip diameter of approximately 80 nm. A semiconductor analyzer (4156C, Agilent, Santa Clara, CA, USA) was employed for the electrical characterization. A voltage was applied to one of the probe tips while the other probe tip was grounded. In general, the voltage sweeps were performed from 0 V→4.5 V→0 V→−4.5 V→0 V with a voltage step width of 0.0225 V under high vacuum conditions (10^−6^ mbar). For each particle, two consecutive current–voltage characteristics (I–V curves) were recorded. The electrical conductivity was derived from the recorded I–V curves. The normality of the measured data was tested applying the Shapiro–Wilk outlier test [48]. According to the Shapiro–Wilk test, six out of the 19 investigated particles were determined as outliers. The mean value and the standard deviation of the electrical conductivity were calculated excluding these outliers. For purposes of comparison, the local electrical conductivity measurement was also performed on LMO particles. A more detailed description of the measurement setup and procedure can be found in the Supplementary Materials and reference [30].

To verify the crystal structure and the chemical composition of the LNMO particles investigated by the local electrical transport measurement, selected area electron diffraction (SAED) and EDX were performed on individual LNMO particles in the above-mentioned TEM. The simulation of the theoretical electron diffraction patterns and the assignment of the electron diffraction patterns were performed using the software JEMS-SAAS [49]. These analyzed LNMO particles were subjected to local electrical conductivity measurement and the electrical conductivities of these particles were determined. Due to the high complexity to conduct the measurements on exactly the same particles both in TEM and in SEM, the measurements were only successfully performed on two particles so far. For purposes of comparison, SAED was also performed on LMO particles.

To examine the effect of Ni doping of LMO materials on their conductivity via the small-polaron hopping mechanism, first principles calculations were performed on both the undoped LMO and the doped stoichiometric LiNi_0.375_Mn_1.625_O_4_ system. For these purposes, spin polarized calculations were performed using the Vienna ab initio Simulation Package (VASP) [50,51], in which the core–valance electron interactions were treated using the projector augmented wave (PAW) formalism [52,53]. The valence electrons considered for each kind of atom were Li(2s^1^2p^0^), O(2s^2^2p^4^), Mn(3d^6^4s^1^) and Ni(3d^9^4s^1^), in which the electrons were treated using the semi-local PBE functionals [54]. To avoid the interaction between the two images of the polaron due to periodic boundary conditions, we modeled the cells using a large 56-atom supercell derived from the spinel structure. A plane-wave cutoff energy of 480 eV was employed on a 7 × 7 × 7 Monkhorst–Pack k-point mesh using Gaussian smearing. The most stable relaxed configuration for each of the systems were ascertained after the change of free energy of the supercell was less than 10^-4^ eV. To account for strong on-site Coulomb repulsion for the 3D electrons of the Mn and Ni atoms, the Hubbard parameter U was added to the GGA functional in the rotationally invariant approach [55], in which only the difference (U_eff_ = U−J) between the Coulomb repulsion U and screened exchange J parameters must be specified. In the present work, we chose U_eff_ = 4.5 eV for both Mn and Ni, as these values have been shown to produce reasonably good estimates in previous studies [36,37,38]. The calculated lattice constants and bond lengths were benchmarked thoroughly against results from previous studies and are provided in the Supplementary Materials.

### 2.3. Cathode Preparation and Cell Assembly

The cathode sheets with LMO and LNMO powders were prepared as reported in reference [45]. The active material-containing slurry was prepared by mixing and ultrasonically dispersing 81.6 wt % of as-prepared LNMO, 10.4 wt % of super C 65 carbon (IMERYS Graphite & Carbon, Düsseldorf, Germany) and 8.0 wt % of polyvinylidene fluoride (PVDF, Sigma-Aldrich, Steinheim, Germany) in N-Methyl-2-pyrrolidone (NMP, Sigma-Aldrich). It was coated on aluminum foil (Western Plastics, Calhoun, GA, USA) using a Mayer rod with a wet thickness of 50 µm. Afterwards, the coated foil was dried at 100 °C overnight, punched out with a diameter of 16 mm and transported into an argon-filled glove box. For the electrochemical tests, ECC-Std test cells (EL-Cell GmbH, Hamburg, Germany) were assembled in an argon-filled glove box with LMO or LNMO cathodes, lithium metal (ø = 18 mm, Albemarle Corporation, Charlotte, NC, USA) as anode, 100 µL 1 M LiPF_6_ (Sigma-Aldrich) in 1:1 (*w*:*w*) mixture of ethylene carbonate (EC, Sigma-Aldrich) and diethyl carbonate (DEC, Sigma-Aldrich) as electrolyte and a glass fiber separator (ø = 18 mm, *t* = 260 µm, Whatman, Maidstone, UK). The configuration of the test cell is illustrated in Figure S1 in the Supplementary Materials.

### 2.4. Electrochemical Characterization

The electrochemical tests were performed on a Basytec LAB battery tester (Basytec GmbH, Asselfingen, Germany).

The electrochemical behavior of the as-prepared LNMO sample was characterized by CV. The cell potential ranged from 3.5 to 5.0 V at a scan rate of 0.05 mV/s.

The specific discharge capacity of the as-prepared LNMO was measured by galvanostatic cycling between 3.5 and 5.0 V with a constant current of C/20 for 10 cycles. The C-rate was calculated with the theoretical capacity of LiNi_0.5_Mn_1.5_O_4_, i.e., 146.7 mAh/g. With the experimentally measured specific discharge capacity, the C-rates were recalculated for further galvanostatic cycling and rate capability tests.

To characterize the cycling stability of the as-prepared LNMO, galvanostatic cycling was performed. After two formation cycles with C/20, the test cells were charged and discharged between 3.5 and 5.0 V with C/2 for 100 cycles.

The rate performance of the as-prepared LMO and LNMO samples was analyzed by galvanostatic cycling with various C-rates (C/20–20C). The cut-off voltages for LMO and LNMO samples are 3.5–4.5 V and 3.5–5.0 V, respectively. The discharge current was varied from C/20 to 20C, while the maximal charge current was set as C/2. For each C-rate, the test cells were cycled for five cycles.

## 3. Results and Discussion

### 3.1. Crystal Structure and Particle Morphology of As-Prepared LNMO

**Figure 1:**
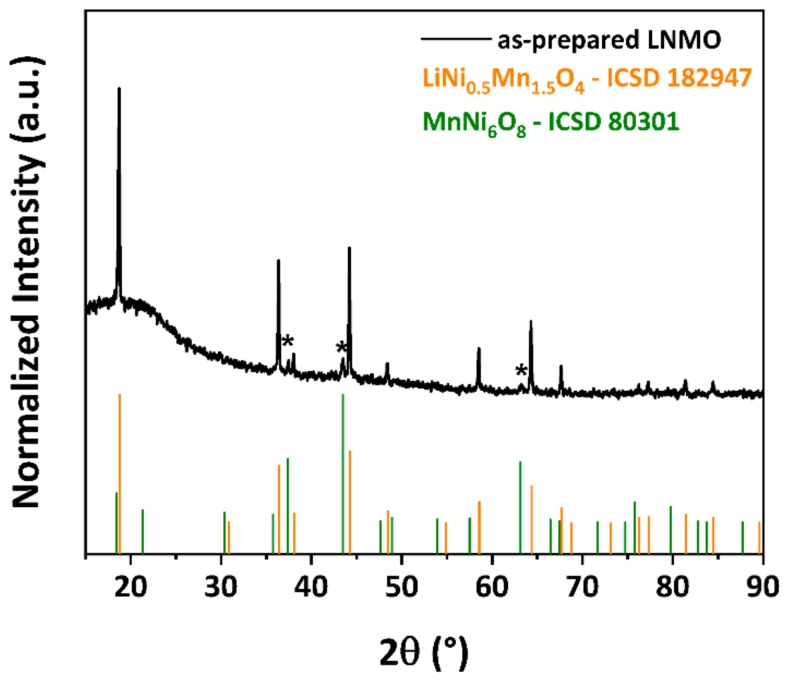
Powder XRD pattern of LNMO prepared via polyol-mediated synthesis.

The crystallographic structure of as-prepared LNMO was analyzed by pXRD. The obtained pXRD pattern is illustrated in Figure 1. The majority of the diffraction peaks can be indexed to cubic spinel structure with the space group of Fd$\bar{3}$m. Weak reflections at 2θ = 37.5°, 43.4° and 63.6° (marked as * in Figure 1) were observed, attributed to a rock salt MnNi_6_O_8_ secondary phase, which is reported in the literature as a common impurity phase in LNMO materials [56,57,58]. As the impurity phase is electrochemically inactive within the potential window investigated in this paper, the presence of this phase would lead to lower specific capacity of the as-prepared LNMO, as it was also included in the calculation of the active material [58]. The broad diffraction peak observed within 15–25° is a measurement artifact caused by our experimental set-up.

Previous studies revealed that the crystallographic structure of LNMO is strongly influenced by the preparation methods, especially by the annealing conditions. Samples synthesized at temperatures higher than 700 °C exhibit the disordered phase, whereas prolonged annealing at 700 °C favors the formation of the ordered phase [16,20,59]. Kunduraci et al. [24] showed that the ordered/disordered phase transition is driven by the oxygen deficiency in the spinel and is accompanied by the formation of a rock salt secondary phase. The ordered/disordered phase can be also tuned by precise control of the cooling rate immediately after calcination at high temperature, which determines the amount of oxygen uptake for the LNMO at around 700 °C [22,60]. Since the as-prepared LNMO was calcined at 800 °C for 24 h with a slow cooling process, we suggest that an oxygen- and Ni-deficient disordered structure was dominant in the as-prepared LNMO, where short-range Ni/Mn ordering could be expected [22,60].

**Figure 2:**
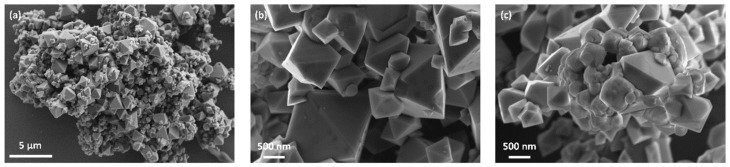
SEM micrographs of as-prepared LNMO: (**b**,**c**) the particle morphologies of the octahedral particles and the particles with irregular shape, respectively, with higher magnification than (**a**).

The particle size and morphology of the as-prepared LNMO were analyzed by SEM. Figure 2 shows the SEM micrographs of the as-prepared LNMO. As can be seen in Figure 2a,b, most of the particles in the sample exhibited octahedral shape with the particle size of 0.5–3 µm, whereas a small fraction of octahedral particles with the size of about 100–200 nm can be observed as well. According to Chemelewski et al. [59,61], the octahedral particles consist of {111} family of planes on the particle surface. In addition, particles with irregular shape are also visible, as illustrated in Figure 2c.

**Figure 3:**
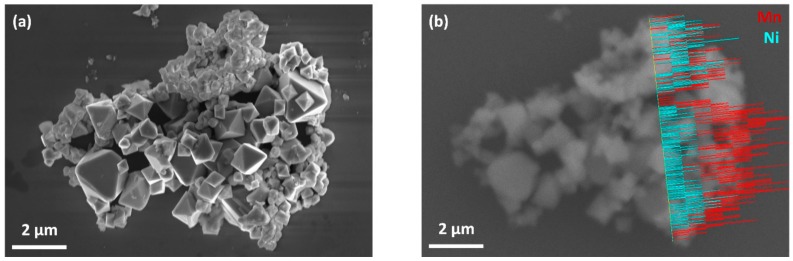
SEM micrograph (**a**); and EDX line scan in the same region with Mn in red and Ni in turquoise (**b**) of as-prepared LNMO.

To further investigate the chemical composition of the octahedral particles and the particles with irregular shape, an agglomerate with relatively high amount of particles with irregular shape was selected and an EDX line scan was performed in the region, where particles with both morphologies were observed. As can be seen in Figure 3b, the Mn:Ni intensity ratio of the particles with octahedral shape was approximately 2–3 times higher than that of the particles with irregular shape, which indicates that the Mn:Ni molar ratio was higher in octahedral particles than in the particles with irregular shape. In addition, EDX measurements (Table S1 in the Supplementary Materials) on individual particles in TEM revealed that the particles with irregular shape exhibited significantly lower Mn:Ni molar ratios in comparison to the octahedral particles, which indicates that these particles are richer in Ni content than the octahedral ones. The pXRD results discussed above confirmed that cubic spinel phase and secondary MnNi_6_O_8_ phase coexisted in the as-prepared LNMO. Therefore, it is assumed that octahedral particles exhibited the spinel phase, whereas the secondary MnNi_6_O_8_ phase was dominant in the particles with irregular shape.

### 3.2. Electronic Charge Transport Properties of As-Prepared LNMO

The electrical conductivity of individual LNMO particles was analyzed by local electrical transport measurement using a nanorobotics setup in an SEM. During the measurements, I–V curves were recorded on 19 individual particles with octahedral shape. The electrical conductivities of these particles were derived from the I–V curves measured by the local electrical transport measurements. Further details on the process for determination of the electrical conductivity are given in the Supplementary Materials.

An I–V curve of one individual LNMO particle is plotted exemplarily in Figure S3 in the Supplementary Materials. The individual LNMO particles exhibited nonlinear I–V curves, which is characteristic for semiconductors [62]. It has to be noted that the nonlinearity of the recorded I–V curves may also be attributed to the potential barrier at the interface between the metallic probe tips and the LMO particle, i.e., the Schottky barrier [63]. The experimental conditions, i.e., the process of particle addressing, the contact pressure, etc., were kept constant during each measurement, so that interfacial effects, such as the Schottky barrier, should be the same in all measurements and were thus not further evaluated.

After evaluation under the Shapiro–Wilk test, data from six out of 19 measured particles were excluded for the calculation of the mean value and the standard deviation of the electrical conductivity. The electrical conductivity of the as-prepared LNMO was thus determined as (1.1 ± 0.8) × 10^−4^ S/cm, which is about one order of magnitude higher than the electrical conductivity values of LNMO materials reported in the literature (1.7 × 10^−5^ − 3.2 × 10^−6^ S/cm) [24,25,26]. The difference between the electrical conductivity values from this work compared with those reported in the literature is due to the different measurement setups.

For purposes of comparison, the electrical conductivity of as-prepared LMO was also determined. It exhibited an electrical conductivity of (1.3 ± 1.0) ×·10^−3^ S/cm, which was about one order of magnitude higher than the electrical conductivity of the individual LNMO particles. Previous work revealed that the electronic conduction mechanism in both LMO and LNMO materials is small-polaron hopping [26,64]. As the polaron states are localized at the Mn^3+^ sites, the electronic conductivity of the materials is assumingly determined mainly by the Mn^3+^ content in the material. A lower Mn^3+^ content in the spinel would thus result in a lower electrical conductivity. As the average oxidation state of Mn in the spinel increases upon Ni substitution, it is suggested that the as-prepared LNMO will exhibit lower amount of Mn^3+^ than the as-prepared LMO. On this basis, a lower measured electrical conductivity for the as-prepared LNMO in comparison to the as-prepared LMO can be rationalized. In agreement with this hypothesis, Molenda et al. observed a decrease of electronic conductivity with increasing Ni content in the Ni-doped LMO materials as well [26].

It must be noted that the electronic conductivity of LNMO materials has been reported to be influenced by its crystallographic structure as well [24,25]. The electronic conductivity of disordered LNMO materials can be as much as 2.5 orders of magnitude higher than that of ordered LNMO materials [24,25]. Nevertheless, the influence of the crystallographic structure on the electronic conductivity was significantly more pronounced for LNMO materials with dominantly ordered structure than for those with dominantly disordered structure [25]. Moreover, the lower electronic conductivity of ordered LNMO materials was attributed to their lower Mn^3+^ content than the disordered LNMO materials [24,25]. Accordingly, we suggest that the electronic conductivity of LNMO materials with dominantly disordered structure is mainly determined by its Mn^3+^ content.

To investigate the crystallographic structure and chemical composition of the LNMO particles with octahedral shape, individual octahedral particles were selected in the SEM (as exemplarily shown in Figure S4 in the Supplementary Materials). These individual octahedral particles were subjected to SAED and EDX in the TEM. Afterwards, they were transported into the SEM and local electrical transport measurements were performed. Due to the high complexity involved in the chain of processes necessary for conducting the measurements on exactly the same particles both in TEM and in SEM, the measurements were only successfully performed on two particles so far.

**Figure 4:**
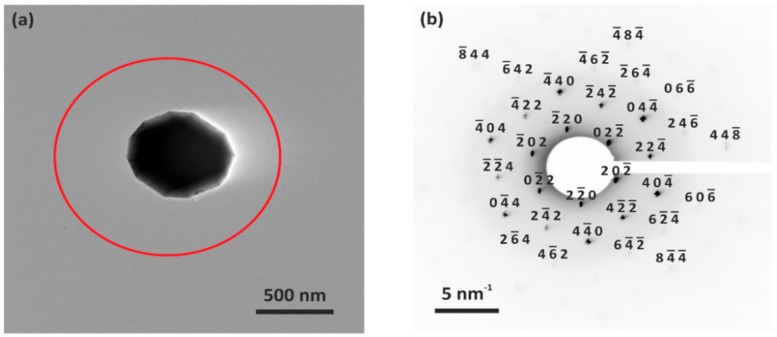
SAED pattern of as-prepared LNMO: (**a**) TEM micrograph of individual LNMO particle; and (**b**) inverted SAED pattern measured in the region of the red-circled area in (**a**).

A TEM micrograph and a measured SAED pattern of one of the individual octahedral LNMO particles are exemplarily depicted in Figure 4.

As exemplarily shown in Figure 4b, periodically arranged diffraction spots were measured for both individual particles in SAED. This indicates that the particles were single crystalline. Due to the thickness of the particles, only the lower order diffraction spots could be observed. The diffraction spots in Figure 4b can be indexed to the {111} zone axis of cubic spinel LiNi_0.5_Mn_1.5_O_4_ (ICSD No. 182947) with the space group of Fd$\bar{3}$m. The slight deviation between the measured and theoretical SEAD patterns is due to the different lattice parameter, which may be caused by slight various Ni content in the LNMO. Nevertheless, with contrast enhancement, several additional weak diffraction spots could be observed, which can be assigned to LNMO with the space group of P4_3_32 (see Figure S5 in Supplementary Materials) [17]. This indicates that the investigated LNMO particle showed signature of partial ordering. Similar SAED pattern was observed by Zheng et al. for their LNMO material prepared with a cooling rate of 1 °C/min [22], which was also applied in this work. They revealed that during the slow cooling process (<3 °C/min), oxygen deficiency is reduced by the oxygen intake and short-range Ni/Mn ordering occurs. Nonetheless, since the intensity of the additional diffraction spots was very low, we assume that the structure of our as-prepared LNMO particles was dominantly disordered. The SAED pattern of the other individual LNMO particle is shown in Figure S6 in the Supplementary Materials. Accordingly, it is assumed that all the octahedral LNMO particles were single crystalline with dominantly disordered spinel phase.

SAED was also performed on LMO particles. The obtained electron diffraction pattern is exemplarily shown in Figure S7 in the Supplementary Materials. Similar to the LNMO particles, periodically arranged diffraction spots were measured, indicating that the particles were single crystalline. As reported in reference [45], the as-prepared LMO only exhibited particles with octahedral shape in a size range of 0.5–3 µm. Therefore, we assume that all the octahedral LMO particles were single crystalline.

The Mn and Ni in the individual LNMO particles were analyzed by EDX in the TEM, as listed in Table 1. The stoichiometry of the individual LNMO particles was calculated based on the EDX results assuming the Li and O contents in the stoichiometry are 1 and 4, respectively. In both investigated particles, a Ni stoichiometry smaller than 0.5 was measured. This indicates that the individual LNMO particles were Ni-deficient, which resulted from the formation of the Ni-rich secondary phase and is in accordance with the pXRD results.

These analyzed individual octahedral LNMO particles were subjected to the local electrical transport measurement in the SEM. The determined electrical conductivities of these individual LNMO particles lay in the range of the measured electrical conductivities of the 19 LNMO particles described above, which validated that our local electrical measurements indeed revealed the intrinsic properties of the as-prepared LNMO spinel.

To examine the effect of Ni doping of LMO materials on their conductivity, first principles calculations were performed on both the undoped LMO and the doped LiNi_0.375_Mn_1.625_O_4_ system, which was stoichiometrically very similar to the system obtained from our experimental analysis. The mechanism of the electronic conduction in LMO has been proposed to be in form of the hopping of a Jahn–Teller (JT) small-polaron [64], which is transferred across the structure in form of distortions in the Mn-O bonds surrounding the polaron-carrying Mn^3+^ ions.

Undoped LMO is known to exhibit cubic spinel structure at room temperature, which undergoes phase transition to an orthorhombic phase at around 230 K [35]. In the present work, however, the LMO system was optimized with an orthorhombic Fddd structural model, which has been argued to be the right theoretical strategy in several previous studies [36,65]. The room temperature cubic phase results from the existing Mn^3+^/Mn^4+^ disorder, which is captured very poorly by DFT calculations on such systems. The stoichiometric LMO supercell of 56 atoms considered in this work consisted of 8 Li, 16 Mn and 32 O atoms, resulting in a theoretical 8:8 ratio of Mn^3+^/Mn^4+^.

The LiNi_0.375_Mn_1.625_O_4_ supercell, on the other hand, was modeled with a the cubic disordered Fd$\bar{3}$m phase, which is known to be the most stable phase for a similar LiNi_0.5_Mn_1.5_O_4_ system [38,39] and was also verified by SAED to be dominant in our as-prepared LNMO. The stoichiometric LiNi_0.375_Mn_1.625_O_4_ supercell of 56 atoms considered in this work consisted of 8 Li, 3 Ni, 13 Mn and 32 O atoms. Assuming an ideal +2 oxidation state of Ni ions, the Mn^3+^/Mn^4+^ ratio would ideally be 2:11, which implies a significant drop in the polaron carrier density within the unit cell as compared to that of LMO. Achieving a perfectly disordered phase is computationally not possible within the framework of periodic DFT calculations and, therefore, 31 different configurations exploring all possibilities of doping of three Ni atoms at 16 possible positions in the supercell were considered to establish the most stable unit cell configuration. The difference between the most stable and least stable configurations and that between the most stable and the second most stable configurations were found to be approximately 1.1 and 0.1 eV/cell, respectively, both of which were well above the 25 meV room temperature thermal fluctuation limit. These observations validate our choice of the most stable system for further calculations of polaron hopping. Further, it was observed that the distribution of Ni in the supercell was very uniform, corroborating the fact that such a uniform structure would yield the highest configurational entropy, and would thus be most stable [66,67].

Finally, hopping barriers for small-polaron hopping were calculated for both LMO and LiNi_0.375_Mn_1.625_O_4_ using the climbing image Nudged elastic Band method (CI-NEB), which attempts to locate the transition state at a saddle point along the reaction coordinate on the potential energy surface [68]. At first, several configurations for each of the two systems were tried to locate the most stable orientation and location of the polarons in form of JT distortions. Seven intermediate states along the reaction coordinate were considered in our case to capture any of the fine features of the hopping curve or any other metastable intermediate states.

**Figure 5:**
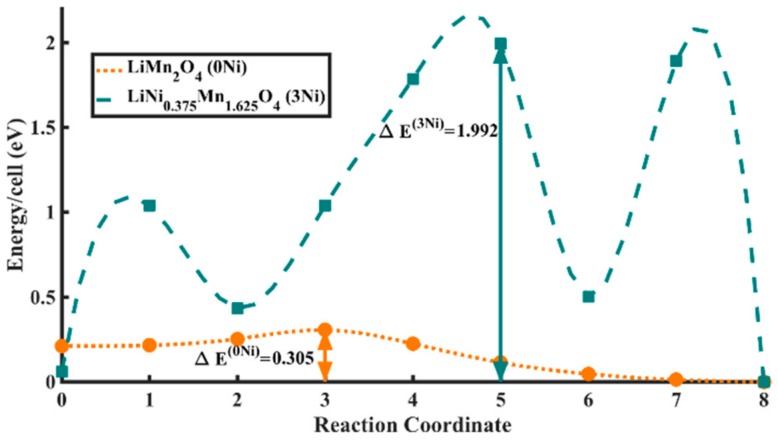
First principles calculated polaron hopping barriers for LMO (peach) and LiNi_0.375_Mn_1.625_O_4_ (teal). The system energy values are normalized and mentioned per supercell. Reaction Coordinate 0 is the initial and 8 is the final stable state for each of the cases.

As can be seen in Figure 5, the barrier for polaron hopping for the undoped case was found to be ΔE(0Ni) = 0.305 eV (Reaction Coordinate 3), which is in excellent agreement with a previous work by Ouyang et al. [37] on the same system. To ascertain oxidation states of Mn atoms, Bader charge analysis was performed, wherein the extent of an atom is determined based on its electronic charge density, which is decided by zero flux surfaces that are used to divide atoms [69]. For Bader charge analysis of the most stable LMO state, Mn atoms were classified as either in +3 or +4 oxidation state, depending on whether they had higher or lower electronic charge than the average charge on Mn atoms. It was clearly observed that eight Mn atoms in the supercell had an average of 0.29 electronic charge more than the other eight Mn atoms. Hence, the calculated stoichiometric ratio between Mn^3+^/Mn^4+^ was 1/1 in LMO, which is what is expected.

**Figure 6:**
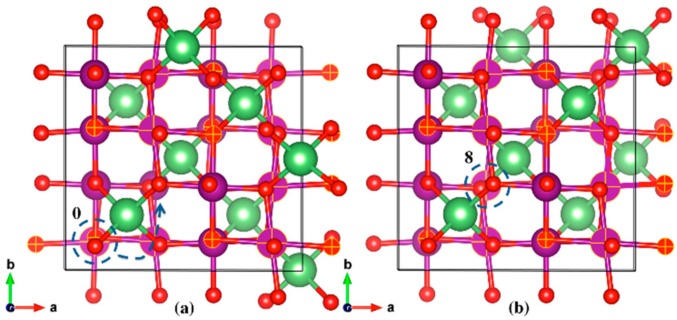
Geometric structures of the initial (**a**) and final (**b**) stable states for LMO polaron hopping. The selected Mn and O atoms, and Mn-O bonds (matte finish) depict the elongated bonds, in this case along the a-axis. The Mn atom marked 0 is the atom centered at the JT distortion in the initial state, which hops to the Mn atom marked 8 in the final stable state. Li (green), Mn (purple), O (red).

The initial and final geometric structures of the two most stable LMO polaron states are shown in Figure 6a,b, respectively. The initial (Reaction Coordinate 0) and final (Reaction Coordinate 8) locations of the JT distortion centered Mn^3+^ ions were also depicted in the same figure. The most stable location of the polarons (Reaction Coordinate 8 in Figure 5 and Figure 6b) was found to have columnar ordering of Mn^3+^ and Mn^4+^ as seen from the JT distortion analysis, which is also in agreement with previous studies [35]. The specific bond lengths for the initial and final states can be found in the Supplementary Materials.

The polaron hopping simulations for LiNi_0.375_Mn_1.625_O_4_ system revealed several unique features. Unlike for the case of LMO, several metastable cases were encountered on the path of hopping, as seen in Figure 5. On careful post-analysis of the geometry at each of the positions along the reaction coordinate, it was confirmed that all new metastable polaron states converged to the nearest stable states along the reaction coordinate. The barrier of hopping for the new polaron state was found to be ΔE(3Ni) = 1.992 eV (Reaction Coordinate 5), which is six-fold higher than that for LMO.

Unlike LMO, a more continuous distribution of oxidation states was found from Bader charge analysis in this case. Three Mn atoms were found to have an average of 0.33 higher electronic charge than the other ten Mn atoms. Hence, the calculated stoichiometric ratio between Mn^3+^/Mn^4+^ was 3/10 in LiNi_0.375_Mn_1.625_O_4_, which is very close to the ideal value of 2/11. It must be noted that the computational localization of electrons using even the DFT+U approach is not failsafe, and could be the reason for these deviations and continuous charge distribution.

**Figure 7:**
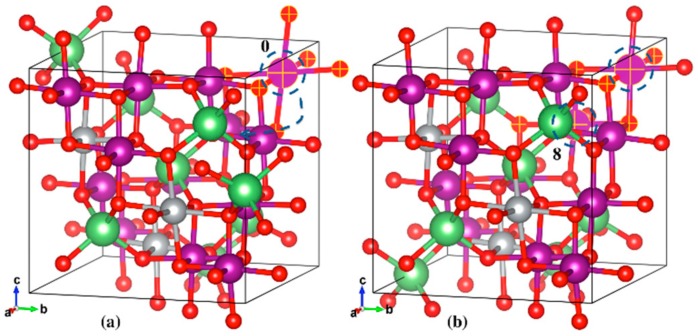
Geometric structures of the initial (**a**) and final (**b**) stable states for LiNi_0.375_Mn_1.625_O_4_ polaron hopping. The selected Mn and O atoms, and Mn-O bonds (matte finish) depict the elongated bonds, in this case along all axes. The Mn atom marked 0 is the atom centered at the JT distortion in the initial state, which then hops to and is shared with the Mn atom marked 8 in the final stable state. Li (green), Ni (silver), Mn (purple), O (red).

The initial and final geometric structures of the two most stable LiNi_0.375_Mn_1.625_O_4_ polaron states are shown in Figure 7a,b, respectively. The initial state shown in Figure 7a had six elongated Mn-O bonds, all on one single Mn atom (Reaction Coordinate 0). The slightly more stable final state had the JT distortion centered on two different Mn atoms, with the other atom (Reaction Coordinate 8) encircled in Figure 7b. The specific bond lengths for the initial and final states can be found in the Supplementary Materials.

It must be noted, however, that the DFT band gap for the undoped system was found to be 0.39 eV and for the doped system it was found to be 0.58 eV, which cannot preclude the possibility that band conduction might have a role in determining the electrical conductivity of the two systems. DFT calculations are, however, known to severely underestimate band-gaps even with the Hubbard U corrections, which implies that the contribution of band conduction in both cases will probably be equally negligible. In any case, our simulations clearly indicated that the scarcity of Mn^3+^ in the doped case would lead to a decrease in its electronic conductivity via small-polaron hopping as compared to that of the undoped system.

### 3.3. Electrochemical Performance of As-Prepared LNMO

**Figure 8:**
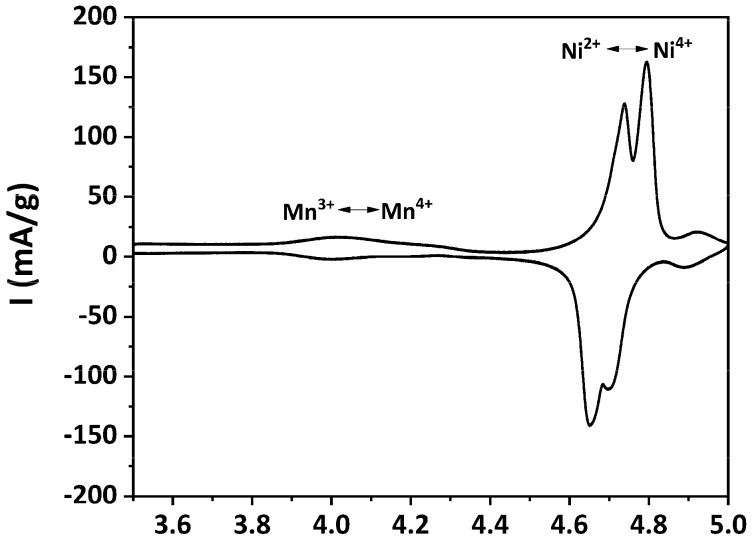
Cyclic voltammogram of as-prepared LNMO.

The electrochemical properties of the as-prepared LNMO were evaluated by CV. The obtained cyclic voltammogram is plotted in Figure 8.

Two pairs of well-resolved, sharp redox peaks were recorded at around 4.7 V vs. Li/Li^+^, corresponding to the Ni^2+^/Ni^3+^ and Ni^3+^/Ni^4+^ redox processes [9,70]. The potential difference between the oxidation and the reduction peaks (ΔE_p_) for the Ni^2+^/Ni^3+^ and Ni^3+^/Ni^4+^ redox processes were determined as 0.09 V and 0.10 V, respectively. These very small ΔE_p_s indicate fast Li^+^ extraction/insertion kinetics in the as-prepared LNMO [23]. A pair of small redox peaks, which is attributed to the Mn^3+^/Mn^4+^ redox process, can also be observed at about 4 V vs. Li/Li^+^. This indicates that a small amount of Mn^3+^ was present in the as-prepared LNMO, which is in agreement with the pXRD results. Another minor pair of redox peaks can be observed at approximately 4.9 V vs. Li/Li^+^, the origin of which is still unclear. Similar voltammograms were also reported by Yang et al. [71]. They attributed these redox peaks at about 4.9 V vs. Li/Li^+^ to decomposition of the electrolyte. Caballero et al. observed an oxidation peak at about 5.1 V and ascribed this peak to the release of oxygen from the spinel framework [72]. According to previous studies [9,17,24,73], the features of the obtained cyclic voltammogram indicates that the as-prepared LNMO exhibited dominantly the disordered structure, which is in accordance to the SAED result.

**Figure 9:**
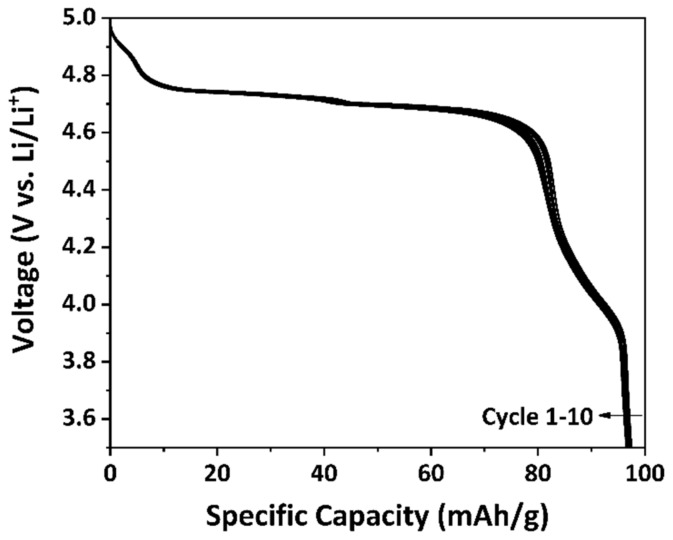
Discharge curves of the as-prepared LNMO with a constant current of C/20.

The specific discharge capacity of the as-prepared LNMO was determined by galvanostatic cycling between 3.5 V and 5.0 V with a constant current of C/20 for 10 cycles. The C-rate was calculated with the theoretical capacity of LiNi_0.5_Mn_1.5_O_4_, i.e., 146.7 mAh/g. Figure 9 illustrates the discharge curves of the as-prepared LNMO. At the 10th cycle, the as-prepared LNMO exhibited a specific discharge capacity of around 97 mAh/g. As revealed by the pXRD result, MnNi_6_O_8_ impurity phase was present in the as-prepared LNMO material, which is electrochemically inactive [58] in the investigated potential window but was included in the calculation of the active material. Thus, the specific discharge capacity of the as-prepared LNMO material is lower than the values reported in the literature [11,22,40,41]. Two voltage plateaus were observed at around 4.7 V vs. Li/Li^+^, which are attributed to the Ni^2+^/Ni^3+^ and Ni^3+^/Ni^4+^ redox processes [70]. A small voltage shoulder at around 4 V vs. Li/Li^+^ could be observed as well, which is contributed by the Mn^3+^/Mn^4+^ redox process [24]. The presence of a voltage shoulder at around 4 V vs. Li/Li^+^ indicates that the as-prepared LNMO exhibited a certain amount of Mn^3+^, which is in accordance with the pXRD and the CV results. The Mn^3+^ percentage contents (p(Mn^3+^)_4V_) in the sample can be indicated by the ratio between the discharge capacity recorded between 4.5 V and 3.5 V and the total discharge capacity of the 2nd cycle [25]:

p(Mn^3+^)_4V_ = (Q_3.5–4.5V_/Q_total_) × 100%

The p(Mn^3+^)_4V_ of the as-prepared LNMO was characterized as 15.1%.

**Figure 10:**
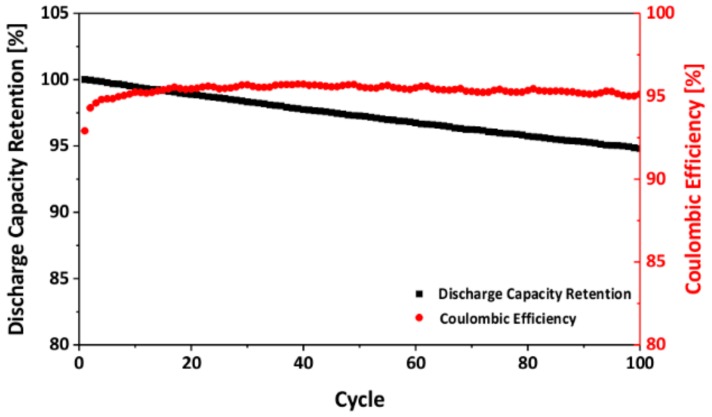
Cycling stability of the as-prepared LNMO.

The cycling stability of the as-prepared LNMO samples was characterized by galvanostatic cycling. Figure 10 illustrates the discharge capacity retention of the as-prepared LNMO over 100 cycles. After 100 cycles, it exhibited a capacity retention of 95%, indicating a very stable cycling behavior. The coulombic efficiency of the sample increased in the first several cycles, which was probably due to electrolyte decomposition and solid electrolyte interface (SEI) formation [74]. Afterwards, it remained constant at approximately 95%. This reveals that there was an irreversible capacity loss on each cycle, which might be attributed to the decomposition of the electrolyte and the formation of an unstable SEI layer [74].

Previous investigations revealed that the capacity fading observed in LNMO materials is probably due to the Mn and Ni dissolution, which results from the electrochemical oxidation of the electrolyte at voltages above 4.2 V vs. Li/Li^+^ [75,76,77]. When Mn^3+^ is present in the material, Mn disproportionation may occur as well, which leads to further Mn dissolution and thus capacity fading.

Nevertheless, the cycling stability of LNMO is not only determined by the presence of Mn^3+^. Recent computational [78,79] and experimental [59,61,80,81] studies revealed that LMO and LNMO materials with octahedral shape and {111} family of planes on the surface showed superior cycling stability to the other particle morphologies. As described in the SEM section above, our as-prepared LNMO exhibited octahedral shape. Therefore, we suggest that the high stability of the as-prepared LNMO might be accredited to its particle morphology, which preponderated the negative influence of Mn^3+^.

**Figure 11:**
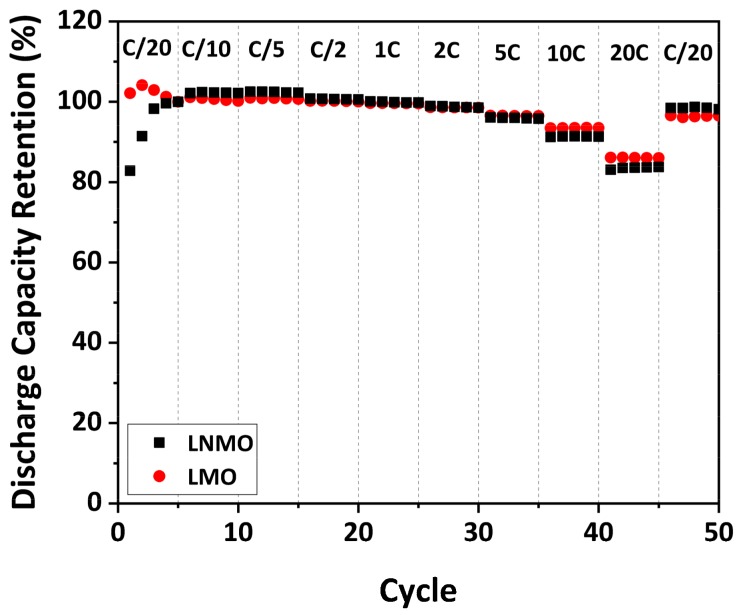
Rate capability of as-prepared LMO and LNMO.

To study the rate capability of the as-prepared LNMO, galvanostatic cycling at various C-rates was performed. For purposes of comparison, the as-prepared LMO was also investigated. It must be noted that due to the difference in specific capacity of the as-prepared LMO and LNMO, the discharge capacity retention instead of the specific discharge capacity is illustrated to avoid misleading information. The discharge capacity retention was determined as the ratio of the discharge capacity at a certain cycle to the one at the 5th cycle at C/20, taking into accounts the SEI formation during the first several cycles. The discharge capacity retention of the as-prepared LMO and LNMO at various C-rates is plotted in Figure 11.

The as-prepared LNMO exhibited 100% discharge capacity retention at low C-rates (C/20–C/2). When the C-rate was increased from C/2 to 5 C, it showed discharge capacity retentions larger than 95%. At high C-rates (10 C and 20 C), it delivered high discharge capacity retention of 91% and 84%, respectively. When the C-rate was reduced to C/20, the as-prepared LNMO exhibited a discharge capacity retention of 98%, indicating that the irreversible damage to the material caused by the fast discharge was negligible. The result is comparable to the results of disordered LNMO materials with various particle sizes and morphologies reported in the literature [19,20]. Similar to cycling stability, the rate capability of LNMO materials with octahedral shape and {111} family of planes on the surface was also reported to be superior to that of materials with other particle morphologies [59,61,80]. Therefore, we suggest that the favorable surface orientation of the as-prepared LNMO could be supportive for the observed high rate capability.

Comparing the as-prepared LNMO to the as-prepared LMO, it appears that the discharge capacity retentions of the as-prepared LMO at high C-rates are, in most instances, only slightly higher than the ones of the as-prepared LNMO. Taking the measurement error in account, this difference is not significant. This indicates that the rate capability of LMO is similar to that of LNMO, although the local electrical transport measurements revealed an order of magnitude higher electronic conductivity for LMO than for LNMO. A recent study from Moorhead-Rosenberg et al. [20] challenges the belief that the Mn^3+^ content is responsible for the rate capability of LNMO materials. They emphasized that Mn^3+^ is only present in the (almost) fully lithiated spinel. For most of the charge/discharge cycles, charges are carried by Ni ions (Ni^2+^, Ni^3+^ and Ni^4+^). Our result supports their argumentation to the extent that the rate capability of LNMO and LMO materials is not limited by the electronic conductivity of the fully lithiated materials, which is mainly influenced by the Mn^3+^ content in the materials. To investigate whether the electronic conductivity of the materials during charging and discharging is related to the rate capability, local electrical transport measurements on partially and fully delithiated LNMO materials should be performed in the future.

## 4. Conclusions

In this work, we successfully prepared Ni-doped LiMn_2_O_4_ spinel via polyol-mediated synthesis and investigated its electronic charge transport properties and electrochemical performance. The as-prepared LNMO showed dominantly disordered structure and exhibited advanced cycling stability as well as rate capability. Local electrical transport measurements on individual particles demonstrated that the electrical conductivity of the as-prepared LNMO was about one order of magnitude lower than that of the undoped LMO. The DFT calculations also indicated that the energy barrier for polaron hopping in LNMO was much higher than that in LMO, which could be possibly related to the scarcity of available Mn^3+^ due to Ni doping. Nevertheless, similar rate capability was observed for both LMO and LNMO materials, which challenges the belief that the Mn^3+^ content is responsible for the rate capability of LNMO materials. Our work demonstrated the significance of investigations on the individual particle level on the micro- and nanometer scale and offers new insights into the intrinsic properties of LNMO. It should be pointed out that our experimental results give no indication about the Mn^3+^ content and the charge status change of Mn during cycling, which was predicted by DFT calculations for only completely lithiated states. This would require operando X-ray Absorption Spectroscopy measurements at different state of charge accompanied by DFT calculation results, which will be part of our future works.

## Figures and Tables

**Table 1:** Stoichiometry of individual LNMO particles based on EDX measurements in TEM.

Particle	Mn Content (At %)	Ni Content (At %)	Stoichiometry
1	81	19	LiNi_0.38_Mn_1.62_O_4_
2	80	20	LiNi_0.40_Mn_1.60_O_4_

## Supplementary Materials

The following are available online at http://www.mdpi.com/1996-1944/11/5/806/s1, Figure S1: Cell configuration of an ECC-Std test cell designed by EL-Cell GmbH; Figure S2: Scheme of the experimental setup (a); exemplary SEM micrograph shows an individual particle addressed by two probe tips (b); Figure S3: Exemplary I–V curve recorded on one individual LNMO particle; Figure S4: Exemplary SEM micrograph of an individual LNMO particle; Figure S5: Inverted SAED pattern of LNMO particle 1 with contrast enhancement. Additional weak diffraction spots, which can be assigned to LNMO with space group of P4_3_32, could be observed; Figure S6: SAED pattern of as-prepared LNMO (particle 2). (a) TEM micrograph of individual LNMO particle; (b) inverted SAED pattern measured in the region of the red-circled area in (a) The diffraction spots can be indexed to the [111] zone axis of cubic spinel LiNi_0.5_Mn_1.5_O_4_ (ICSD No. 182947) with the space group of Fd$\bar{3}$m; (c) inverted SAED with contrast enhancement. Additional weak diffraction spots, which can be assigned to LNMO with space group of P4_3_32, could be observed; Figure S7: SAED pattern of as-prepared LMO. (a) TEM micrograph of individual LMO particle; (b) inverted SAED pattern measured in the region of the red-circled area in (a). The diffraction spots can be indexed to the [323] zone axis of cubic spinel Li_1.09_Mn_1.91_O_3.99_ (ICSD No. 55738) with the space group of Fd$\bar{3}$m; Table S1: Stoichiometry of individual particles with different particle morphologies based on EDX measurements in TEM.
