# Supplementary material for: Electrochemical and Electronic Charge Transport Properties of Ni-Doped LiMn2O4 Spinel Obtained from Polyol-Mediated Synthesis
Source: Materials (Basel). 2018 May 16;11(5):806. doi: 10.3390/ma11050806 (PMC5978183; doi:10.3390/ma11050806)
Supplement: Supplementary file 1 [file materials-11-00806-s001.pdf]

materials

Supplementary Information

Electrochemical and Electronic Charge Transport Properties of Ni-doped $LiMn_2O_4$ Spinel Obtained from Polyol-Mediated Synthesis

Shuo Yang [1,2]*, Dirk Oliver Schmidt [1,2], Abhishek Khetan [3], Felix Schrader [1,2], Simon Jakobi [1,2], Melanie Homberger [1,2], Michael Noyong [1,2], Anja Paulus [2,4], Hans Kungl [2,4], Rüdiger-Albert Eichel [2,4,5], Heinz Pitsch [3] and Ulrich Simon [1,2,]*

[1] Institute of Inorganic Chemistry, RWTH Aachen University, Aachen, 52074, Germany oliver.schmidt@rwth-aachen.de (D.O.S.); felix.schrader@rwth-aachen.de (F.S.); simon.jakobi@ac.rwth-aachen.de (S.J.); melanie.homberger@ac.rwth-aachen.de (M.H.); michael.noyong@ac.rwth-aachen.de (M.N.)
[2] Jülich Aachen Research Alliance-JARA, Jülich, 52428, Germany
[3] Institute for Combustion Technology, RWTH Aachen University, Aachen, 52056, Germany; askhetan@gmail.com (A.K.); h.pitsch@itv.rwth-aachen.de (H.P.)
[4] Institute of Energy and Climate Research (IEK-9: Fundamental Electrochemistry), Forschungszentrum Jülich, Jülich, 52425, Germany; anj.paulus@fz-juelich.de (A.P.); h.kungl@fz-juelich.de (H.K.); r.eichel@fz-juelich.de (R.-A. E.)
[5] Institute of Physical Chemistry, RWTH Aachen University, Aachen, 52074, Germany
* Correspondence: shuo.yang@rwth-aachen.de; Tel.: +49-241-80-99388; ulrich.simon@ac.rwth-aachen.de; Tel.: +49-241-80-94644

Received: 18 April 2018; Accepted: 14 May 2018; Published: date

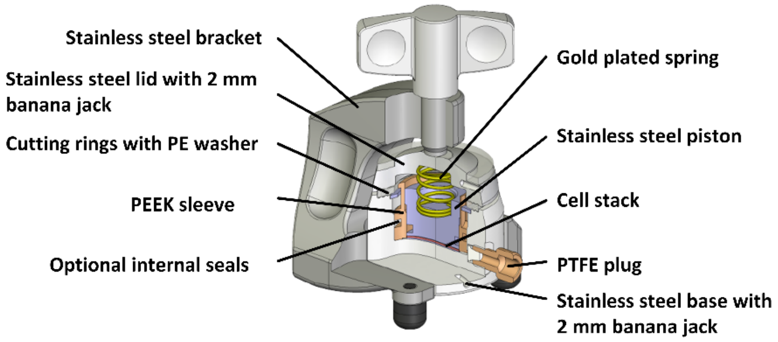

Figure S1. Cell configuration of an ECC-Std test cell designed by EL-Cell GmbH.

Table S1. Stoichiometry of individual particles with different particle morphologies based on EDX measurements in TEM.

Particel	Particle Morphorlogy	Mn Content (atom%)	Ni Content (atom%)	Mn:Ni (atom:atom)
1		80	20	4.00
2		81	19	4.26
3		76	24	3.17
4		81	19	4.26
5		78	22	3.55
6	octahedral	85	15	5.67
7		80	20	4.00
8		85	15	5.67
9		80	20	4.00
10		78	22	3.55
11		76	24	3.17
12		26	74	0.35
13		19	81	0.23
14		20	80	0.25
15		20	80	0.25
16	irregular	34	66	0.52
17		21	79	0.27
18		36	64	0.56
19		17	83	0.20
20		19	81	0.23

Local Electrical Transport Measurement

The local electrical transport measurements were performed in a ZEISS LEO Supra 35 VP SEM equipped with a nanorobotics system (Klocke Nanotechnik) and a 4156C semiconductor analyzer (Agilent). The nanorobotics system allows electrical contact of samples on the micro- and nanometer scale with up to four probe tips mounted to four absolute positioning manipulators. Detailed information about the setup is described elsewhere [1].

Homemade metallized atomic force microscopy (AFM) tips were utilized as probe tips for the measurements. The AFM tips (ATEC-NC) were purchased from Nanosensors. They were

isotropically coated with an 80/20 Pt/Ir alloy by radio frequency sputtering (0.017 mbar Ar, 40 W) using a sputtering system (model Classic 250, Pfeiffer Vaccum GmbH). Prior to each measurement, the probe tips were freshly prepared and examined in the SEM to exclude contaminations and/or damage of the tips. The radii of the probe tips were measured in the SEM as well. Typically, the probe tips exhibit a radius of curvature of approximately 80 nm.

The electric conductivity of the probe tips was controlled before the measurements by contacting the two tips with each other. A voltage sweep from -10 mV→10 mV→-10 mV was applied to the tips with a current constrain of 1 μA. Experiments were only conducted with the tips, when a linear I-U behavior was observed and the tip-tip resistance was below 1000 Ω. Typically, the tip-tip resistance is around 300-600 Ω.

For the local electrical transport measurements, LNMO particles were deposited on TEM grids with SiO_2 windows (SiO_2 thickness: 20 nm, SiMPore Inc.). Prior to the measurements, the particles were analyzed in the TEM to verify the crystal structure and the chemical composition of the particles.

During the measurement, 19 individual LNMO particles were addressed with two probe tips, as shown exemplarily in **Figure S2**. A voltage was applied to one of probe tips while the other probe tip was grounded. The voltage sweeps were performed from 0 V→4.5 V→0 V→−4.5 V→0 V with a voltage step width of 0.0225 V under high vacuum conditions (10^{-6} mbar). For each particle two consecutive current-voltage characteristics (I-V curves) were recorded. The electrical conductivity σ of the investigated particle was derived from the recorded I-V curves according to:

$$\sigma = \frac{I}{U}$$

where U = ±0.315 V is the applied voltage. I is the current measured at the applied voltage, d is the tip-tip distance measured in the SEM images and A is contact area of the two probe tips, which is estimated in this work as $1.6 \cdot 10^{-10}$ cm^{-2}. A voltage of ±0.315 V was chosen since IS measurement of a pressed pellet was conducted at a similar voltage (0.3 V) in the literature [2].

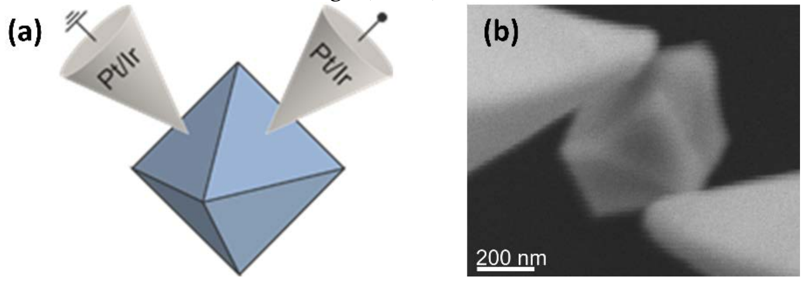

Figure S2. Scheme of the experimental setup (a); exemplary SEM micrograph shows an individual particle addressed by two probe tips (b).

The normality of the measured data was tested applying the Shapiro-Wilk outlier test [3]. As criteria W(95%) as well as W(99%) were applied. In general, for each particle, eight electrical conductivity values were obtained from two consecutive measurements. These values were processed by the Shapiro-Wilk outlier test and outlier were determined according to the test. Afterwards, the mean value of the electrical conductivity was calculated for each particle, neglecting the previously detected outliers. The calculated mean values were subsequently processed by the test and outliers were determined. Hence, the mean electrical conductivity values of these particles were disregarded. Finally, the mean value of the electrical conductivity values and the standard deviation of the mean values of all particles were determined for each sample, excluding the outlier values. For the LNMO measurement series, six out of the 19 investigated particles were determined according to the Shapiro-Wilk outlier test as outlier and were not included in calculation of the mean electrical conductivity.

For purposes of comparison, local electrical transport measurements were also performed on 27 LMO individual particles. Four out of 27 particles were determined according to the Shapiro-Wilk outlier test as outlier and were not included in the calculation of the mean electrical conductivity.

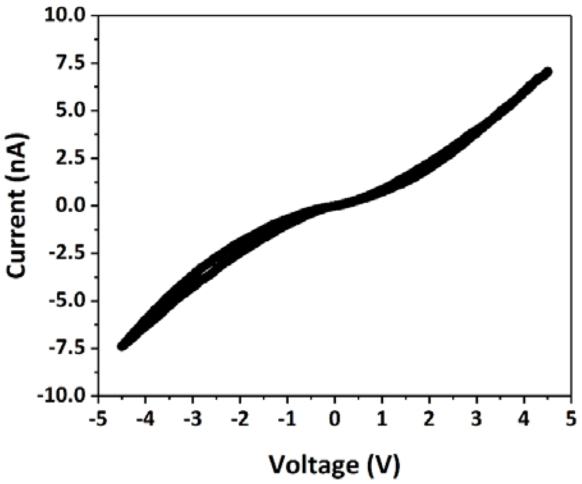

Figure S3. Exemplary I-V curve recorded on one individual LNMO particle.

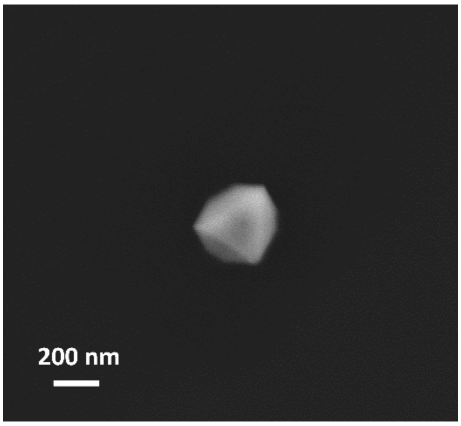

Figure S4. Exemplary SEM micrograph of an individual LNMO particle.

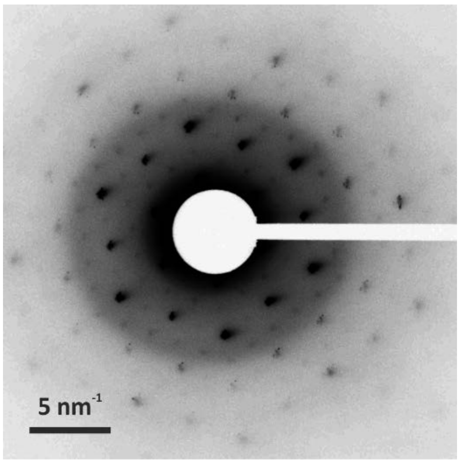

Figure S5. inverted SAED pattern of LNMO particle 1 with contrast enhancement. Additional weak diffraction spots, which can be assigned to LNMO with space group of $P4_332$, could be observed.

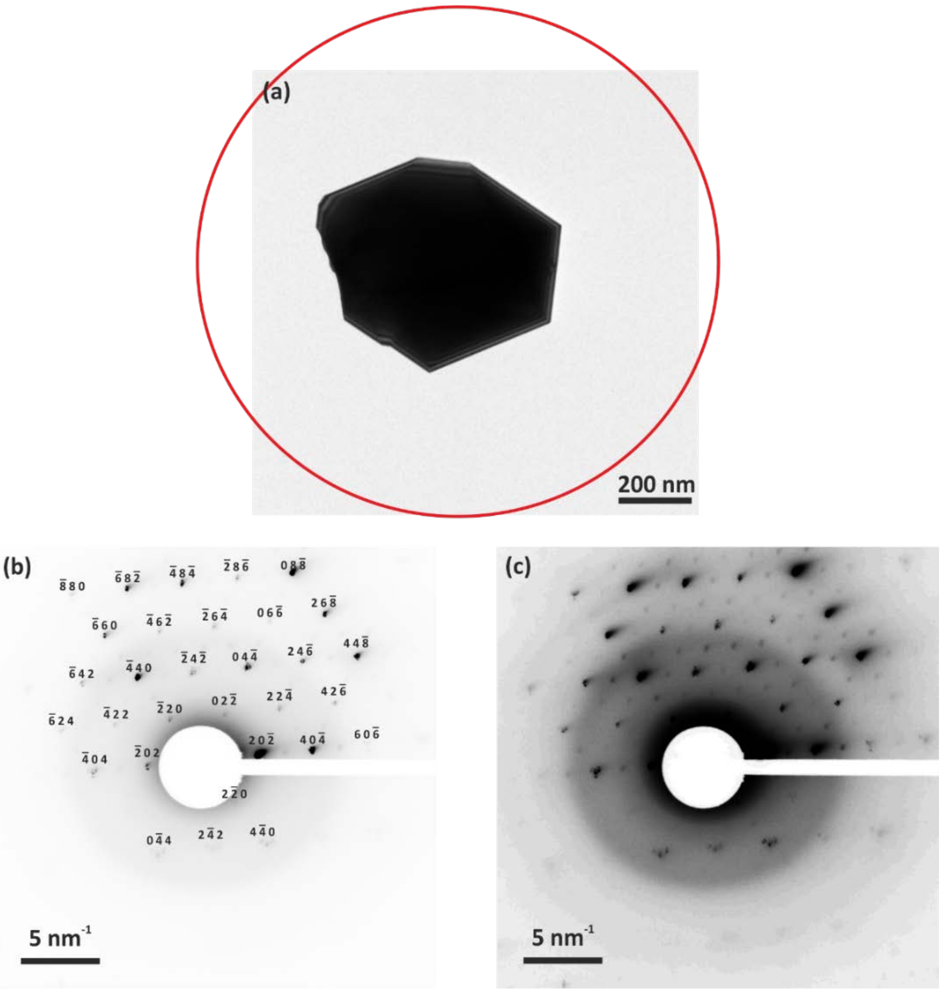

Figure S6. SAED pattern of as-prepared LNMO (particle 2). (a) TEM micrograph of individual LNMO particle; (b) inverted SAED pattern measured in the region of the red-circled area in (a) The diffraction spots can be indexed to the [111] zone axis of cubic spinel $LiNi_{0.5}Mn_{1.5}O_4$ (ICSD No. 182947) with the space group of $Fd\bar{3}m$; (c) inverted SAED with contrast enhancement. Additional weak diffraction spots, which can be assigned to LNMO with space group of $P4_332$, could be observed.

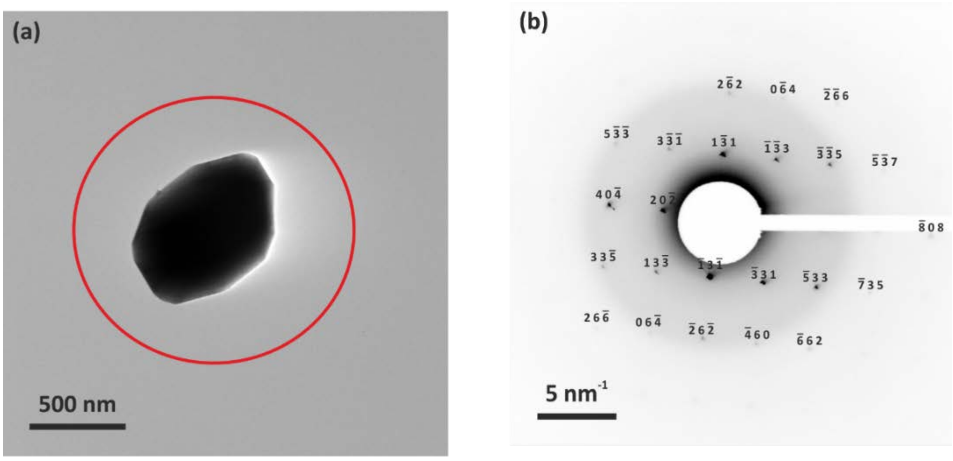

Figure S7. SAED pattern of as-prepared LMO. (a) TEM micrograph of individual LMO particle; (b) inverted SAED pattern measured in the region of the red-circled area in (a). The diffraction spots can be indexed to the [323] zone axis of cubic spinel $Li_{1.09}Mn_{1.91}O_{3.99}$ (ICSD No. 55738) with the space group of $Fd\bar{3}m$.

First principles calculated data

First principles calculated data for LMO:

- 8 Li, 16 Mn, 32 O atoms in supercell
- Lattice parameters (orthorhombic): a = 8.614 Å, b = 8.160 Å, c = 8.117 Å
- Mn-O bond length changes during polaron hopping, with significant changes (> 0.1 Å) highlighted:

Mn Atom	Figure	State	Mn-O Bond direction and lengths (Å)					
			+x	-x	+y	-y	+z	-z
0	5a	initial	2.164	2.143	2.039	1.971	1.942	1.930
8	5a		1.966	1.946	1.941	1.878	1.919	1.933
0	5b	final	1.945	1.945	1.924	1.923	1.922	1.922
8	5b		2.167	2.167	1.961	1.962	1.950	1.950

- Bader Charges on Mn atoms in the most stable LMO structure:
  Average oxidation state: 1.902
  Standard deviation: 0.15

Bader Calculated Mn Oxidation State	Theoretical Mn oxidation state	Average
1.742	3	1.757
1.745	3	
1.748	3	
1.748	3	
1.748	3	
1.771	3	
1.777	3	
1.778	3	
2.028	4	2.047
2.036	4	
2.044	4	
2.045	4	
2.049	4	
2.056	4	
2.056	4	
2.062	4	

First principles calculated data for $LiNi_{0.375}Mn_{1.625}O_4$:

- 8 Li, 3 Ni, 13 Mn, 32 O atoms in supercell
- Lattice parameters (cubic): a = b = c = 8.205 Å
- Mn-O bond length changes during polaron hopping, with significant changes (> 0.1 Å) highlighted:

Mn Atom	Figure	State	Mn-O Bond direction and lengths (Å)					
			+x	-x	+y	-y	+z	-z
0	6a	Initial	2.135	2.135	2.135	2.135	2.135	2.135
8	6a		1.949	1.901	1.894	1.938	1.921	1.910
0	6b	Final	2.151	2.155	1.988	1.966	1.978	1.922
8	6b		1.957	1.908	2.111	2.109	2.035	1.978

- Bader Charges on Mn atoms in the most stable $LiNi_{0.375}Mn_{1.625}O_4$ structure:
  Average oxidation state: 1.696
  Standard deviation: 0.175

Bader Calculated Mn Oxidation State	Theoretical Mn oxidation state	Average
1.441	3	1.621
1.502	3	
1.550	3	
1.591	3	
1.592	3	
1.594	3	
1.701	3	
1.714	3	
1.730	3	
1.795	3	
1.886	4	1.946
1.913	4	
2.039	4	

References

1. Noyong, M.; Blech, K.; Rosenberger, A.; Klocke, V.; Simon, U. *In situ* nanomanipulation system for electrical measurements in SEM. *Meas. Sci. Technol.* **2007**, *18*, N84-N89, DOI: 10.1088/0957-0233/18/12/N02.
2. Kunduraci, M.; Amatucci, G.G. Synthesis and Characterization of Nanostructured 4.7 V $Li_xMn_{1.5}Ni_{0.5}O_4$ Spinels for High-Power Lithium-Ion Batteries. *J. Electrochem. Soc.* **2006**, *153*, A1345, DOI: 10.1149/1.2198110.
3. Kaiser, R.E.; Mühlbauer, J.A. *Elementare Tests zur Beurteilung von Meßdaten: Soforthilfe für statististische Tests mit wenigen Meßdaten*; Bibliographisches Institut, 1983; ISBN: 9783411057740.

© 2018 by the authors. Submitted for possible open access publication under the terms and conditions of the Creative Commons Attribution (CC BY) license (http://creativecommons.org/licenses/by/4.0/).
